# Optimizing Strategies for Developing Genetically Encoded Voltage Indicators

**DOI:** 10.3389/fncel.2019.00053

**Published:** 2019-02-26

**Authors:** Madhuvanthi Kannan, Ganesh Vasan, Vincent A. Pieribone

**Affiliations:** ^1^The John B. Pierce Laboratory, New Haven, CT, United States; ^2^Department of Cellular and Molecular Physiology, Yale School of Medicine, Yale University, New Haven, CT, United States; ^3^Department of Neuroscience, Yale School of Medicine, Yale University, New Haven, CT, United States

**Keywords:** genetically encoded voltage indicators, ArcLight, *Ci*VSD, voltage sensitivity, membrane potential

## Abstract

Genetically encoded optical indicators of neuronal activity enable unambiguous recordings of input-output activity patterns from identified cells in intact circuits. Among them, genetically encoded voltage indicators (GEVIs) offer additional advantages over calcium indicators as they are direct sensors of membrane potential and can adeptly report subthreshold events and hyperpolarization. Here, we outline the major GEVI designs and give an account of properties that need to be carefully optimized during indicator engineering. While designing the ideal GEVI, one should keep in mind aspects such as membrane localization, signal size, signal-to-noise ratio, kinetics and voltage dependence of optical responses. Using ArcLight and derivatives as prototypes, we delineate how a probe should be optimized for the former properties and developed along other areas in a need-based manner. Finally, we present an overview of the GEVI engineering process and lend an insight into their discovery, delivery and diagnosis.

## Introduction

To understand the brain is to understand how individual neurons process information – all the way from the tips of dendrites to the soma, and onward to the nerve terminals. The real challenge, however, is to decode information processing in every neuron in the context of its neighbors or, in other words, intact neural circuits because neuronal interactions form the basis of sense perceptions, behavior and consciousness.

In mammals, neural ensembles consisting of thousands of neurons are often involved during a complex process such as reconstructing a visual scene or maneuvering in the local environment. To decode information processing during such behaviors, it becomes necessary to obtain simultaneous electrical recordings of input–output activity patterns from all participating neurons at single-cell resolution.

Conventional electrophysiology relies on the use of patch electrodes (intracellular) and multi-electrode arrays (MEAs) (extracellular) to measure electrical events that accompany neuronal signal processing. Patch electrodes can measure both subthreshold synaptic potentials as well as action potentials (APs) but can only record from a handful of neurons at a time. MEAs, on the other hand, consist of hundreds of electrical contacts, and can be used to obtain voltage recordings from several hundred neurons simultaneously. Current extracellular probes can allow for nearly a thousand densely spaced individual recording sites, providing a high spatial resolution for electrical measurements (Jun et al., 2017). However, parsing signals from individual neurons is not straightforward, as most sites sample signals from multiple neurons. Besides, the technique does not allow for targeted recordings in specific cells, cell types or subcellular compartments (Buzsaki, 2004; Spira and Hai, 2013).

The limitations of electrode-based techniques have largely been mitigated by optical approaches to monitoring neuronal activity, such as using synthetic dyes and genetically encoded fluorescent indicators. Using the latter, it is now possible to simultaneously record unambiguously from as many as 500 neurons in three-dimensional space, in awake and behaving animals, while preserving cellular resolution with multiphoton laser scanning modalities (Huber et al., 2012; Katona et al., 2012). Genetically encoded indicators can be engineered to selectively target distinct cell types and subcellular compartments, and to do so at precise stages of development. Genetically encoded calcium and voltage indicators [GECIs and genetically encoded voltage indicators (GEVIs), respectively] enable longitudinal imaging of activity in labeled neurons (Denk et al., 1996; Mainen et al., 1999; Mao et al., 2008; Tian et al., 2009; Mittmann et al., 2011; Chen et al., 2012; Wachowiak et al., 2013; Dana et al., 2014; Rothermel and Wachowiak, 2014; Zhu et al., 2014; Lur et al., 2016), and are the method of choice for functional imaging in subcellular compartments, e.g., dendritic spines inaccessible to electrodes, and organisms less-suited for patch-clamp experiments, such as *Drosophila* and *Caenorhabditis elegans* (Tian et al., 2009; Akerboom et al., 2012; Yoshihara, 2012; Kovacevic et al., 2013).

## Comparison of Genetically Encoded Calcium and Voltage Sensors

Since their development in the late 1990s, fluorescent activity indicators have seen extensive improvements in sensitivity and kinetics (Miyawaki et al., 1997; Romoser et al., 1997; Gong et al., 2015; Chamberland et al., 2017; Kannan et al., 2018; Piatkevich et al., 2018), and are beginning to complement electrode-based studies to understand fundamental aspects of neurophysiology.

Calcium indicators are molecular fusions of a calcium sensor, such as calmodulin or troponin C, and a fluorescent reporter protein (FP). GECIs are cytosolic proteins, which, upon binding intracellular Ca^2+^, undergo conformational changes that modulate the fluorescence output of the FP. In some instances, a conformational change in the calcium-binding domain modulates instead the fluorescence resonance energy transfer (FRET) between a pair of donor and acceptor fluorophores. Modern GECIs include the GCaMPs and RCaMPs, GECOs, and Cameleons (Nagai et al., 2004; Palmer et al., 2006; Tian et al., 2009; Zhao et al., 2011; Akerboom et al., 2013; Chen et al., 2013). Because they detect intracellular Ca^2+^, whose levels fluctuate during APs, GECIs provide an indirect measure of suprathreshold neuronal activity.

In contrast, GEVIs are fluorescent transmembrane proteins that can directly sense voltage fluctuations across the membrane during activity and synaptic transmission. The voltage-sensing domain (VSD) in GEVIs may be derived from transmembrane proteins such as ion channels, voltage-sensitive phosphatases or microbial opsins, and is fused to a single FP or a FRET-FP pair (Jin et al., 2012; Gong et al., 2013, 2014; Flytzanis et al., 2014; Hochbaum et al., 2014; St-Pierre et al., 2014; Zou et al., 2014; Piao et al., 2015; Abdelfattah et al., 2016). Opsin-based probes can also function as standalone GEVIs that report activity by virtue of their native albeit dim fluorescence (Gong et al., 2013; Flytzanis et al., 2014; Hochbaum et al., 2014).

**FIGURE 1:**
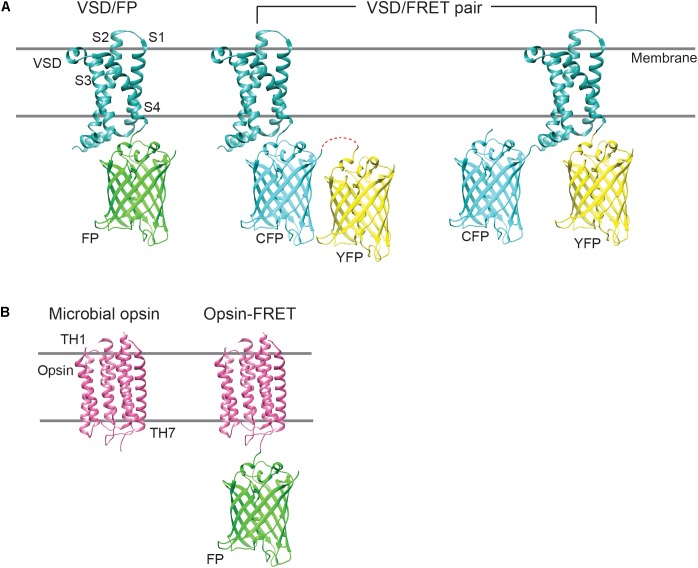
Cartoon showing the different GEVI designs. **(A)** The majority of type 1 GEVIs consist of a molecular fusion of an isolated VSD, derived from a voltage-sensitive phosphatase, and a single FP, or a FRET FP pair. In FRET constructs, the FPs may be fused in tandem or to either terminus of the VSD. CFP, cyan fluorescent protein; YFP, yellow fluorescent protein. **(B)** Type 2 GEVIs comprise standalone microbial opsins, which possess seven transmembrane helices (TH1–TH7), or opsin-FRET constructs, where the opsin may be fused to a fluorophore. Microbial opsins exhibit broad absorption spectra (500–700 nm) and serve to “quench” the fluorescence of the appended fluorophore, during voltage fluctuations.

Genetically encoded voltage indicators exhibit voltage-dependent fluorescence change via one of two distinct mechanisms: (1) by drawing on a conformational change in the voltage sensor or (2) by coupling with active state transitions during the photocycle of pigment retinal, the chromophore in voltage-sensitive opsins. GEVIs that fall under the type 1 category include ArcLight, VSFPs, ASAP1/2, Bongwoori and FlicR1 (Dimitrov et al., 2007; Akemann et al., 2012; Jin et al., 2012; St-Pierre et al., 2014; Piao et al., 2015; Abdelfattah et al., 2016), whereas those part of the type 2 category comprise derivatives of *Archaerhodopsin* (Arch), such as QuasArs, Archers, Archons, and FRET-opsin indicators, namely, QuasAr- mOrange2, MacQ-mCitrine, and Ace2N-mNeon, among others (Flytzanis et al., 2014; Gong et al., 2014, 2015; Hochbaum et al., 2014; Zou et al., 2014; Piatkevich et al., 2018). The different GEVI templates are illustrated in Figure 1.

As sensors of membrane potential, GEVIs are not limited to detecting APs unlike calcium indicators but can also report hyperpolarizations, subthreshold depolarizations such as those associated with synaptic transmission and sustained depolarizations that represent transient activated states. Further, while calcium indicators exhibit slow kinetics (∼50–75 ms for the fast GECI GCaMP6f) and can only resolve spikes at or below 20 Hz (Chen et al., 2013), GEVIs can provide superior temporal resolution, capable of detecting spikes at frequencies greater than 100 Hz (Gong et al., 2015).

## Innovating a Genetically Encoded Voltage Indicator

Owing to their distinctive subcellular localization and mechanism of action, the criteria to engineer voltage indicators are inherently different from calcium indicators, spurring unique challenges in their design, development, and testing.

Voltage indicators require robust trafficking signals to ensure adequate and exclusive membrane expression, a prerequisite for high signal-to-noise ratios (SNR) in optical recordings – the ratio of the peak of optical response to the standard deviation of baseline fluorescence (*F*_0_). Precise subcellular targeting is especially crucial for voltage indicators because useful voltage signals emerge but from a limited area of the cell (the membrane) as compared to the large volume of cytoplasm available for calcium detection.

Likewise, enhancing the sensitivity of voltage indicators is particularly challenging. The sensitivity of a probe per unit voltage change is expressed as the fraction of fluorescence change (Δ*F*) to baseline fluorescence. Calcium indicators exhibit large sensitivities as the slow kinetics of successive calcium transients allows for cumulative increases in fluorescence response amplitudes. Spike-induced calcium transients have short rise times but prolonged decays. Fast AP trains cause individual transients to summate or, in other words, ride on top of one another (Smetters et al., 1999). Thus, during high frequency AP bursts (>∼20 Hz), GECIs bind the built-up calcium over several hundred milliseconds, much after the underlying spikes have decayed, resulting in long-lasting responses with large Δ*F*/*F*_0_ values. Indeed, the exact kinetics (on and off rates) and dynamic range of sensitivities of an indicator depend on its effective concentration, Ca^2+^-binding affinity, and cellular calcium-buffering properties. Nevertheless, GCaMP variants exhibit fractional fluorescence changes of up to 500% for a train of 10 APs and 1000–1700% for 160 APs in cultured neurons, when in fact their Δ*F*/*F*_0_ values for single APs are merely 10–30% (Tian et al., 2009; Chen et al., 2013). Their large *in vitro* sensitivities further make calcium probes favorable for *in vivo* experiments where they provide high SNRs against background fluorescence and signal attenuation in thick tissue. Voltage fluxes, on the other hand, are extremely rapid – a neuronal AP lasts for less than a few milliseconds. The sensitivity of GEVIs is thus measured as the Δ*F*/*F*_0_ for a single AP. Contemporary GEVIs have modest sensitivities of 40–80% Δ*F*/*F*_0_ in cultured cells for 100 mV depolarization (≈AP amplitude) (Jin et al., 2012; Hochbaum et al., 2014; Piao et al., 2015; Yang et al., 2016; Piatkevich et al., 2018). The responses are further dampened *in vivo* due to background fluorescence and light scattering. To enhance signal sizes by at least an order of magnitude, embarking on high-throughput engineering and testing endeavors becomes inevitable.

As direct sensors of membrane potential, GEVIs can be tailored to be sensitive to hyperpolarization. However, the voltage range across which a probe’s sensitivity spans is variable. In many cases, it can be shifted to more negative potentials by carrying out specific mutations in the VSDs (Dimitrov et al., 2007; Baker et al., 2012; Piao et al., 2015). Full optimization of the voltage-dependence of optical responses requires careful consideration of experimental needs and appropriate engineering of the voltage sensor.

In the following sections, we will review the characteristics of an ideal GEVI and the current standing of probes.

### Membrane Localization

To report changes in cellular membrane potential, voltage indicators must reside in the plasma membrane. They capitalize on the cell’s secretory protein trafficking machinery to be transported here (Lippincott-Schwartz et al., 2000). Secretory proteins that are synthesized and assembled at the endoplasmic reticulum (ER) are exported to the Golgi complex where they undergo maturation. They are eventually packaged into post-Golgi vesicles and translocated along microtubules – fine cylindrical structures that constitute the scaffolding of a cell. Sometimes, misfolded proteins and proteins that tightly bind chaperones (protein-folding assistors) can be entrapped in the ER or Golgi complex, leading to intracellular aggregates. By contributing to voltage-insensitive background fluorescence, these aggregates can dampen the SNR of voltage signals in optical recordings.

**FIGURE 2:**
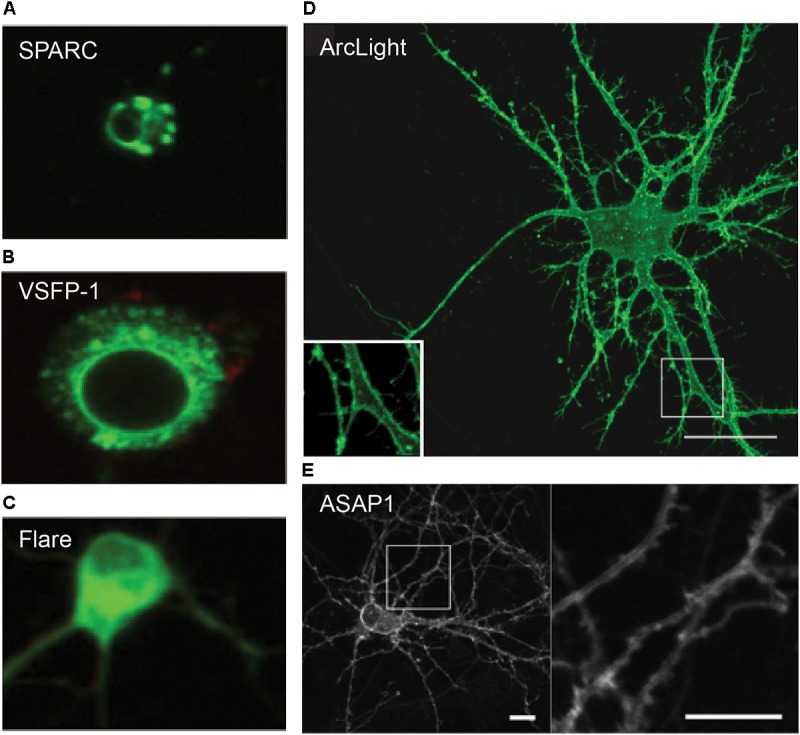
Confocal images of GEVI expression in dissociated hippocampal neurons. **(A–C)** Ion channel-based GEVIs SPARC, VSFP-1 and Flare exhibit poor membrane localization and “web-like” intracellular aggregates, suggestive of protein retention in the ER. **(D,E)** VSD-based voltage sensors Arclight and ASAP1 show excellent membrane expression, which is clearly visible even in subcellular compartments such as dendrites (insets). Scale bars = 10 μm (Baker et al., 2007; Jin et al., 2012; St-Pierre et al., 2014).

The first-generation type 1 GEVIs FlaSh and its derivative Flare, VSFP1 and SPARC, were tandem fusions of a voltage-gated potassium or sodium channel and a single FP or a FRET-FP pair (Siegel and Isacoff, 1997; Sakai et al., 2001; Ataka and Pieribone, 2002; Baker et al., 2007). These GEVIs exhibited intense intracellular aggregation in cultured human embryonic kidney (HEK) cells and hippocampal neurons, with less than 5% fluorescence at the plasma membrane. The intracellular fluorescence often displayed a “web-like” pattern, a classic phenotype suggestive of ER retention (Baker et al., 2007) (Figure 2). Likely due to their poor membrane localization, these early probes exhibited extremely poor voltage sensitivity in neurons both *in vitro* and *in vivo*.

To overcome the issue of aggregation, which was thought to arise from improper folding of multimeric or modular channel proteins, the clunky ion channels of the first-generation GEVIs were replaced by the self-contained, monomeric VSD of a transmembrane phosphatase isolated from the sea squirt *Ciona intestinalis* (abbr. *Ci*VSD) (Dimitrov et al., 2007) (Figure 2). *Ci*VSD is a four-pass transmembrane protein (S1–S4 α helices), which can independently function as a voltage sensor (Murata et al., 2005).

Subsequently, orthologs from other species such as chicken, zebrafish, mouse, human and frog have each been cloned in place of *Ci*VSD as putative voltage sensors. The resultant probes show distinct characteristics, with the chicken (abbr. *Gg*VSD) and zebrafish VSDs (abbr. *Dr*VSD) having profound effects on GEVI kinetics (Baker et al., 2012; Han et al., 2013; St-Pierre et al., 2014).

The majority of VSD-based GEVIs – VSFP2, VSFP-Butterfly, Mermaid, ArcLight, Bongwoori and FlicR1 – contain *Ci*VSD as the voltage sensor fused to a green or red FP, or a FRET-FP pair (Dimitrov et al., 2007; Tsutsui et al., 2008; Akemann et al., 2012; Jin et al., 2012; Piao et al., 2015; Abdelfattah et al., 2016). When the FP or FRET pair is fused to either intracellular terminus of the VSD (S1 or S4), GEVIs show predominant membrane localization in non-neuronal and hippocampal cultures. Membrane localization appears to be impaired when the FP is inserted in the extracellular loop between S3 and S4 domains. This is, however, not the case for a similar FP insertion within *Gg*VSD in the fast voltage sensor ASAP1 (St-Pierre et al., 2014).

Opsin-based indicators have similarly suffered from intracellular aggregation in cultured neurons and brain slices, warranting the need for pixel weighing algorithms for fluorescence response quantification. These algorithms selectively average fluorescence changes across pixels that are informative (membranal) while deemphasizing the contribution from voltage-insensitive background (Hochbaum et al., 2014).

Aside from the voltage sensor, the choice of FP in membrane-targeted fusion proteins also affects membrane expression. Red FPs, such as dsRED, mCherry, and mOrange from the mushroom anemone *Discosoma* (class Anthozoa) have a greater tendency to aggregate, whereas FPs isolated from the jellyfish *Aequorea victoria*, such as the widely used green fluorescent protein (GFP) and its derivatives, generally traffic better (Arrenberg et al., 2009; Gong et al., 2014). Circular permuted (cp) FPs, in which the N- and C-terminal fragments are transposed to make them more permissive to regulation by conformational changes, tend to be more challenging to fold, and voltage probes incorporating cpFPs have a greater propensity to aggregate (Barnett et al., 2012; Abdelfattah et al., 2016).

Appending ER export (FCYENEV) and membrane trafficking (KSRITSEGEYIPLDQIDINV) peptide signals from the inward-rectifier potassium channel K_ir_2.1 to the C-terminus of microbial opsins, has been shown to reduce intracellular aggregates and improve membrane expression in mammalian cells (Gradinaru et al., 2008, 2010). Accordingly, novel GEVIs are engineered to incorporate these peptides as fusion tags (Gong et al., 2014).

Finally, when overexpressing voltage indicators, it is important to carefully evaluate any adverse effects on membrane properties. As GEVIs introduce charge carriers in the membrane, their expression likely affects membrane capacitance – a measure of the charge stored across the membrane – which can in turn affect downstream physiological processes. Capacitive effects of GEVIs are reported to be minimal at expression levels normally achieved. Nevertheless, it is important to thoroughly quantify passive membrane properties and estimate AP widths while developing new indicators (Jin et al., 2012; St-Pierre et al., 2014; Gong et al., 2015).

### Sensitivity

The sensitivity of optical indicators is a critical parameter in functional imaging. As outlined above, it is also the most challenging to improve while engineering GEVIs.

#### The Need for Large Voltage Sensitivity

The optical signal size is an important determinant of a probe’s performance for the following reasons.

First, a probe’s sensitivity directly contributes to the SNR of the optical signals. High SNR is indispensable for reliable data interpretation, including identification of subthreshold events and high-fidelity spike detection. Second, a large sensitivity in preliminary screens with cultured cells ensures that the optical signal is discernible in thick brain slices and *in vivo* experiments, where fluorescence responses are attenuated by tissue absorption, scattering, auto-fluorescence and out-of-focus fluorescence (see section “Signal-to-Noise Ratio”). Third, large optical signals circumvent the need to average responses across multiple sweeps. They hence allow imaging under physiological conditions where responses may vary from trial to trial. A probe that yields a conspicuous signal in single trials allows detection of non-stationary events such as spontaneous APs and AP bursts.

#### Enhancing the Voltage Sensitivity of GEVIs

ArcLight was one of the first described GEVIs to exhibit a large sensitivity – its fluorescence output decreases by as much as 40% Δ*F*/*F*_0_ during 100 mV depolarization steps in HEK cells, 5% in response to APs in cultured neurons and acute brain slices and 2% *in vivo* in the mouse olfactory bulb (Jin et al., 2012; Cao et al., 2013; Kunst et al., 2014; Klein et al., 2015; Storace et al., 2015). ASAPs and Arch-based indicators have also been engineered to exhibit large response amplitudes (Yang et al., 2016; Chamberland et al., 2017; Piatkevich et al., 2018).

##### Targeting the FP or VSD

**FIGURE 3:**
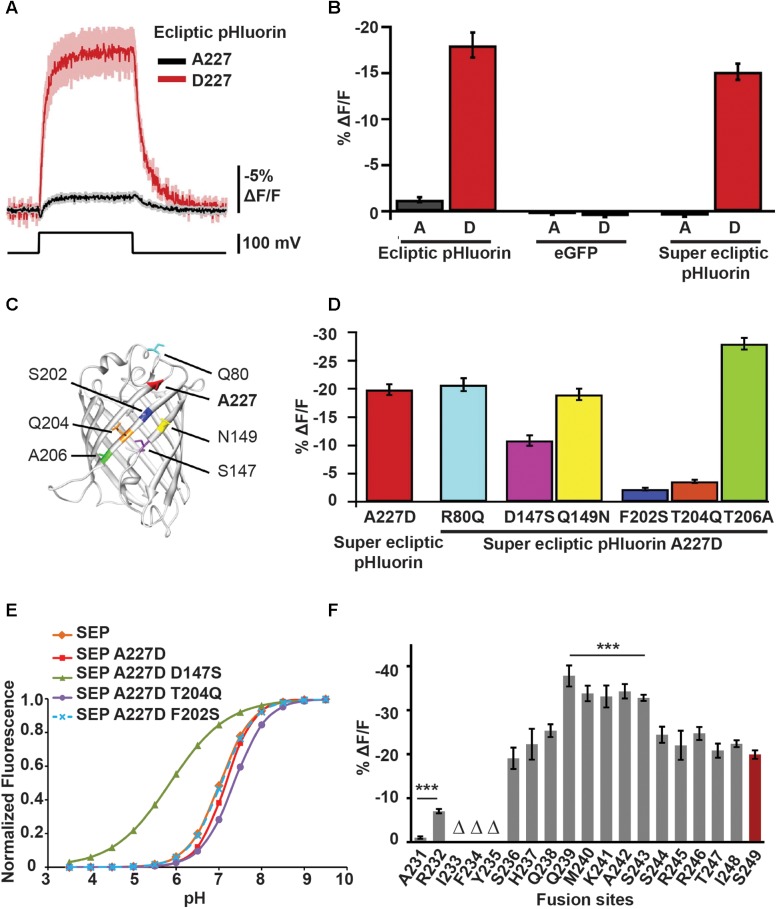
Mutations in the FP sequence as well changes to the *Ci*VSD-FP linker length contribute to ArcLight’s voltage sensitivity. **(A)** Fluorescence responses to 100 mV depolarization of HEK cells transfected with *Ci*VSD-ecliptic pHluorin or *Ci*VSD-ecliptic pHluorin A227D. **(B)** 100 mV step responses (mean ± SEM) of HEK cells expressing 227A or 227D variants of each of *Ci*VSD-ecliptic pHluorin, *Ci*VSD-eGFP or *Ci*VSD-SEP. **(C)** Crystal structure of eGFP (PDB ID 1EMG) highlighting A227 as well as the six most dissimilar residues between SEP and eGFP. The SEP versions are indicated. Note that all four critical residues 147, 227, 202, and 204 reside on the same barrel surface of the fluorophore. **(D)** Fluorescence responses (mean ± SEM) of *Ci*VSD-SEP variants harboring mutations at indicated residues in **(C)**. These mutations serve to replace the SEP amino acids with corresponding residues found in eGFP. **(E)** pH sensitivity curves of SEP and SEP mutants containing eGFP residues at indicated sites. **(F)** Mean response amplitudes to 100 mV depolarization of ArcLight derivatives with modified linker length. Groups that significantly differ from ArcLight S249 are indicated with asterisks. Δ refers to sensors showing poor membrane localization (Jin et al., 2012; Han et al., 2014).

ArcLight’s parental probe was constructed by fusing a pH-sensitive GFP variant, ecliptic pHluorin, to the C-terminus of *Ci*VSD. It exhibited a marginal sensitivity of -1.3% Δ*F*/*F*_0_ per 100 mV depolarization in HEK cells. Subsequently, a serendipitous discovery – an unintended substitution of alanine with aspartic acid at position 227 in the β-barrel of GFP (A227D) – led to an improved variant with a Δ*F*/*F*_0_ of -18% per 100 mV (Jin et al., 2012) (Figure 3A).

Replacing ecliptic pHluorin in ArcLight with superecliptic pHluorin (SEP), which contains the mutations F64L and S65T from enhanced GFP (eGFP), abates the acquired sensitivity but Δ*F*/*F*_0_ can be restored by simply reintroducing 227D (Figure 3B). Importantly, substituting the aspartic acid with any of the other 18 amino acids either completely or partially dampens the optical signal. The other negatively charged amino acid glutamic acid, which has an additional carbon, retains but a third of the improved sensitivity (-6% Δ*F*/*F*_0_ per 100 mV), suggesting that both negative charge *and* reduced steric effects from the compact side chain of aspartic acid are critical for the voltage response (Jin et al., 2012).

In addition to A227D, other residues specific to SEP and/or ecliptic pHluorin seem to play a role in ArcLight’s voltage sensitivity. This is because an ArcLight based on eGFP/A227D, which differs from SEP by nine mutations, fails to produce a voltage signal.

Loss-of-function studies reveal that in fact three mutations in the β-barrel unique to pHluorins – S147D, S202F, and Q204T – are necessary for ArcLight’s voltage-dependent fluorescence response. Introducing these mutations, together with A227D, is sufficient to make an eGFP-based ArcLight as sensitive as the SEP/227D-based sensor (Han et al., 2014) (Figure 3C,D).

However, to date, it remains unclear how these amino acids transduce voltage changes into a fluorescent signal. Some plausible mechanisms have been suggested based on the interaction of the FP with the membrane, pH sensitivity and oligomerization.

Two-photon polarization microscopy of ArcLight-transfected HEK cells suggests that the SEP/227D exhibits linear dichroism, meaning that the fluorophore is likely oriented parallel to the surface of the membrane. This observation together with GFP’s crystal structure data, which shows that all the four critical residues have outward-facing side chains and belong in adjacent β strands (same surface) of the GFP barrel, lead us to speculate that a voltage-dependent conformational change in the *Ci*VSD may swing the FP closer to the membrane and modulate the strength of interactions between the barrel and the membrane. Reversible changes in the strength of these interactions may in turn affect the chromophore environment and its deprotonated (fluorescent) state.

Further, SEP exhibits fluorescence changes as a function of pH (Figure 3E). D147 serves as an intrinsic pH sensor that base-shifts the pH sensitivity curve, bringing the pKa of GFP (the pH at half-maximal fluorescence) to near neutral, the ambient pH of the membrane. It also steepens the slope of the curve (>1). A slope greater than one suggests that even subtle changes in pH, as would be expected to occur during an AP when protons are mobilized by an electric field, can produce a large change in fluorescence. Whether or not ArcLight’s voltage sensitivity indeed arises from SEP’s pH sensing remains elusive. Substitution of other FPs with similar pH curves, such as ratiometric pHluorin or YFP, fails to generate voltage-sensitive variants even in the presence of A227D. It is possible that mutations unique to SEP are crucial for ArcLight’s sensitivity and the absence of these mutations may result in small pH changes being rapidly buffered. Nevertheless, the pH sensitivity of SEP does not appear to solely modulate ArcLight’s responses because mutations in *Ci*VSD can independently tune its sensitivity to different voltage ranges (see section “Voltage Dependence of Optical Responses”).

Lastly, FP oligomerization may also have an impact on voltage sensitivity. Insertion of a tandem SEP downstream of *Ci*VSD dramatically reduces the response size and flips the polarity of the optical signal (+2% Δ*F*/*F*_0_ per 100 mV). In contrast, in the proton-channel-SEP/227D-based indicator Pado, mutation of a putative FP dimerization site diminishes its sensitivity, suggesting that dimerization normally stabilizes the optical signal (Kang and Baker, 2016). It is unclear, however, whether SEP/227D does exist as dimers in intact GEVIs and if so, why dimerization has different effects in different scaffolds.

A more definitive elucidation of the mechanism requires electron microscopy and X-ray crystallization studies to determine the structure of the intact GEVI before and during depolarization. However, membrane proteins are rather challenging to crystallize in their native folded state (Parker and Newstead, 2016).

Likewise, multiple mutations in the opsin core comprising the vicinity of the chromophore have been shown to result in large improvements in voltage sensitivity in Arch-based indicators (Hochbaum et al., 2014; Piatkevich et al., 2018).

The primary sequence of the VSD has also been shown to regulate voltage sensitivity. Recently, a single mutation R415Q in the S4 helix of *Gg*VSD in ASAP uncovered a new variant ASAP2s with strikingly large sensitivity (-38% Δ*F*/*F*_0_ per 100 mV) to depolarizing voltage steps. The response represents a 66% improvement over the parental template ASAP1 (-23% Δ*F*/*F*_0_ per 100 mV) (Chamberland et al., 2017). However, the mutation affected response kinetics, slowing down the rise time from 2.9 to 5.2 ms. This observation is not entirely surprising considering that movements of the S4 helix are speculated to partake in voltage-sensing (see section “Source of VSD”).

Together, these findings point to the important role of the primary amino acid sequence in regulating an indicator’s voltage performance.

##### Targeting the linker

Aside from the VSD and FP, changing the length and composition of the AA sequence in the linker between the two domains has often led to dramatic improvements in voltage sensitivity.

Shortening the linker between the S4 helix of *Ci*VSD and SEP/227D doubled the response amplitude of ArcLight (∼35–40% versus 18% Δ*F*/*F*_0_ per 100 mV). This was achieved by truncating the linker, one amino acid at a time from residue S249 of the VSD, and bringing SEP closer to S4, until the optical response reached saturation viz. between positions S243 and Q239 (Figure 3F).

Similarly, a substitution and a deletion involving two amino acids (A147S ΔA148) in the linker between the S3 helix and FP in ASAP1, enhanced its optical signal to ∼45% from 30% Δ*F*/*F*_0_ per 50 mV, albeit for responses to hyperpolarization (Yang et al., 2016).

Fluorescence resonance energy transfer sensors rely even more on optimal linker lengths (and AA composition) since FRET efficiency is inversely proportional to the sixth power of the distance between donor and acceptor FPs. A type 2 GEVI QuasAr-mOrange2, for instance, was derived by progressively shortening the linker by truncating not only the C-terminus of the voltage sensor cum FRET quencher QuasAr, but also the N-terminus of the FRET donor mOrange2, followed by randomization of two residues at the intersection (Zou et al., 2014).

Although reducing linker lengths in most cases enhances performance by allowing conformational changes in the VSD to better pervade the FP, bringing the two motifs too close to each other can also interfere with their vibrational freedom and dampen the sensitivity. For instance, bringing the FP closer than Q239 of *Ci*VSD diminishes ArcLight’s voltage sensitivity. Moreover, large deletions in the VSD and/or FP can adversely affect protein folding and expression (Han et al., 2014; Zou et al., 2014). We have observed that unconventional FPs, with structures distinct from the classic barrel, tend to require longer linkers. This can be achieved by introducing one or a few randomized (NNK) codons at the junction and selecting the insertional mutants for improved voltage sensitivity.

##### Polarity of the optical signal

Most contemporary GEVIs, except the Arch family of standalone opsins and FlicR1, exhibit a decrease in fluorescence following depolarization. These probes, with negatively sloped voltage-fluorescence relationships (Δ*V*/Δ*F* curves), are brighter at resting conditions than during an AP. Their high baseline fluorescence can be disadvantageous because fluorophores are prone to photobleaching even under ambient illumination intensities, and the loss of fluorescence (reduced photon count) can have a negative impact on SNR. Besides, out-of-focus fluorescence from nearby resting neurons can increase noise levels, further dampening the SNR, in wide-field recordings from intact tissue. For instance, in an *in vivo* olfaction study in the fly, extraneous ArcLight fluorescence from non-coplanar glomeruli contaminated the signals from regions-of-interest (ROIs) comprising the relevant glomeruli on the surface of the antennal lobe. The poor SNR led to odorant responses that were inconsistent with published extracellular recordings in peripheral sensilla. The problem was partially overcome by limiting ArcLight expression in specific glomeruli by genetic targeting (Cao et al., 2013).

Low baseline fluorescence, on the contrary, correlates well with high SNRs, as is the case with the most advanced GCaMP variants (Chen et al., 2013). GCaMPs respond to APs by increasing their fluorescence output compared to resting levels.

Recent work from our lab demonstrates that specific FP mutations can reverse the polarity of the optical signal. Substituting three residues in ArcLight’s SEP with hydrophobic amino acids (D389A, H390A, and Y442V) yields a novel probe with a positively sloped Δ*V*/Δ*F* curve. While 389A serves to flip the polarity, the others, also on the same surface of the β-barrel, enhance the response amplitude in the same direction. Hydrophilic or polar residues at these sites, however, revert the polarity to that of ArcLight. The finding bolsters the argument that voltage-sensitive movements in *Ci*VSD modulate the strength of interactions between the FP and the hydrophobic membrane and suggests that both voltage sensitivity and polarity of optical responses can be altered by targeting the fluorophore (Platisa et al., 2017).

The red voltage indicator FlicR1 also shows a positive response to depolarization (Abdelfattah et al., 2016). FlicR1 consists of *Ci*VSD fused to cp-mApple. It is unclear whether the reversal is due to mApple’s sequence and geometry, which are distinct from the GFP family, or its circular permutation. A previous study has shown that circular permutation can have bidirectional effects on polarity with some *Ci*VSD/cp-eGFP-based scaffolds exhibiting positive and others negative responses to depolarization (Barnett et al., 2012).

##### Implications of signal size for *in vivo* experiments

Their large dynamic range of sensitivities make calcium indicators ideal for routine *in vivo* imaging. In contrast, only a few GEVIs with substantial sensitivities have been demonstrated to work *in vivo* (Cao et al., 2013; Carandini et al., 2015; Gong et al., 2015; Storace et al., 2015; Yang et al., 2016). Low sensitivity is particularly problematic for wide-field (one-photon) microscopy, where out-of-focus fluorescence diminishes SNR (Flusberg et al., 2008; Mukamel et al., 2009).

**FIGURE 4:**
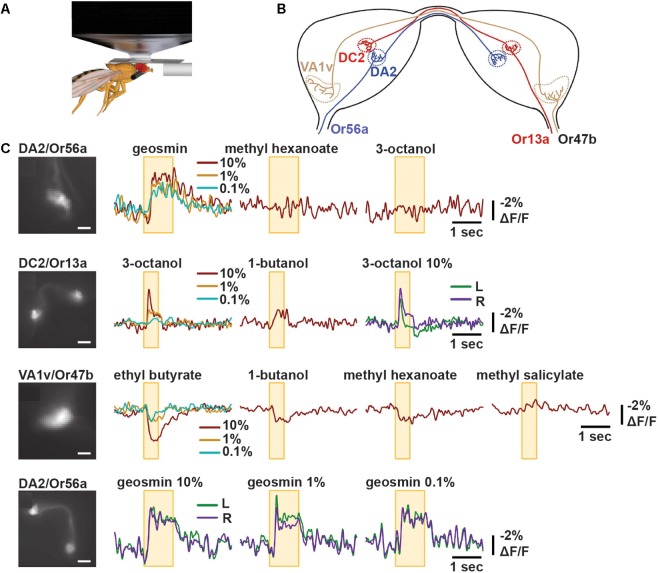
ArcLight reports odorant responses in single trial recordings in *Drosophila*. **(A)** Schematic representation of *in vivo* imaging experiment of *Drosophila* antennal lobe following odorant presentation. **(B)** Schematic diagram of bilateral antennal lobes displaying projections of OSNs expressing Or56a, Or13a, or Or47b to their respective glomeruli. **(C)** Representative optical recordings of membrane activity at presynaptic terminals of OSNs following indicated odor application. The yellow box indicates the time of odor application. The colors indicate the different odorant concentrations 10, 1, or 0.1% or recordings obtained from the left (L) or right (R) glomeruli. Scale bars = 10 μm (panel 2 and 4) and 20 μm (panel 1 and 3) (Cao et al., 2013).

ArcLight was first imaged *in vivo* in *Drosophila*. Here, the probe was genetically targeted to specific olfactory sensory neurons (OSNs) by driving ArcLight expression from promoters of cell-specific olfactory receptor genes. Odorant tuning of presynaptic OSNs and their glomerular projections was studied using wide-field imaging with a blue laser (488 nm). Optical signals were collected at 125 Hz using a low-noise charge-coupled device (CCD) camera. As expected from established electrical recordings of sensilla, the different ArcLight-expressing OSN subtypes exhibited excitatory responses (up to -5% Δ*F*/*F*_0_) to select odorants, with a high degree of odorant tuning. The signals were discernible in single-trial recordings and reported the relative differences in the strength of responses to different odorants and different doses. For instance, the glomerulus DC2 exhibited a stronger response to 3-octanol than 1-butanol (-5% versus -2% Δ*F*/*F*_0_). Additionally, ArcLight also revealed inhibitory responses in VA1v with similar sensitivity (Cao et al., 2013) (Figure 4).

ASAP2f, which was engineered to exhibit a large sensitivity and improved kinetics, has been used to delineate visual response properties in the fly with superior sensitivities of up to -10% Δ*F*/*F*_0_ in averaged sweeps. The data, acquired using a two-photon microscope, provided a high spatial resolution for subcellular imaging (Yang et al., 2016).

Likewise, Ace2N-mNeon, a high-speed FRET-opsin indicator consisting of *Acetabularia* opsin and mNeonGreen, could report orientation tuning properties in sparsely labeled mouse visual cortical neurons with response amplitudes of -3% Δ*F*/*F*_0_ per AP. The probe, which exhibits ∼-10% Δ*F*/*F*_0_ per AP in cultured neurons, has superior kinetics that compensates for the apparent low sensitivity and enhances spike detection rates *in vivo* (Gong et al., 2015).

Although wide-field imaging can dampen the SNR by confounding optical signals with out-of-focus fluorescence, targeting probes to specific cell types can minimize non-specific labeling and improve image quality. Such low-noise recordings are conducive for isolating individual responses in single trials. In addition, they are beneficial for measuring activity in certain cell types, which are inaccessible to electrodes, such as *Drosophila* circadian clock neurons (Cao et al., 2013).

Optical recordings in mice are also facilitated by targeted expression of indicators in genetically identified populations, followed by thinned skull preparations or cranial window implants to enable imaging through intact tissue. Live imaging in mice, however, entails such issues as movement artifacts from breathing and pulsations of blood vessels, hemodynamic artifacts from light absorption (λ < 650 nm) by hemoglobin, and green autofluorescence from endogenous chromophores nicotinamide adenine dinucleotide and flavoproteins. All of the above can essentially contaminate the signals and can be especially problematic when using blue-shifted indicators. Some of these issues can be resolved by engineering red-shifted probes, applying temporal and spatial filtering strategies, or ratiometric imaging with FRET indicators. However, in the long run, enhancing the sensitivity and SNR is key to improving event detection *in vivo.* Indeed, the field saw a huge surge in the use of GECIs after GCaMP3 was described, which then displayed a large enough dynamic range of sensitivities from ∼10 to ∼120% Δ*F*/*F*_0_ for APs and spike detection probabilities that correlate with response sizes (Tian et al., 2012).

### Signal-to-Noise Ratio

The SNR of voltage signals provides a measure of an indicator’s fidelity to detect the incidence (and timing) of individual spikes and subthreshold signals. A large SNR allows for event detection from single trials, eliminating the need for averaging. The SNR of optical recordings depends on intrinsic properties of an indicator such as brightness, quantum yield and excitation/emission wavelengths, as well as extrinsic factors such as protein expression, membrane trafficking, illumination intensity, sources of noise, emission filter, numerical aperture (NA) of the objective and choice of detector.

#### Choice of Fluorescent Protein

An essential first step in engineering a probe is choosing an FP with a high fluorescence quantum yield (φ), brightness, which is the product of φ and extinction coefficient (𝜀), and photostability. FPs such as eGFP, mNeonGreen, mCitrine, and mRuby have high φ (0.5–0.8) and brightness (60–90) and make excellent probes with high SNRs (Jin et al., 2012; St-Pierre et al., 2014; Zou et al., 2014; Gong et al., 2015; Bajar et al., 2016). In contrast, Arch derivatives, which operate as standalone GEVIs, have fluorescence φ of the order of 10^-4^ to 10^-3^. Although opsins can still yield high-SNR signals under high illumination intensities (880 mW mm^-2^ to 8 W mm^-2^, 18–80 times that required for GFP-based probes) and when combined with sensitive electron-multiplying-CCD or complementary metal oxide semiconductor (cMOS) detectors (Flytzanis et al., 2014; Hochbaum et al., 2014), they are impractical for studying intact tissue because high light intensities can cause sample heating, photodamage and tissue autofluorescence. They have since been adapted into opsin-FRET probes where they are fused to standard fluorophores to enable improved detection under standard conditions.

Photostability, defined as the time to photobleach to half-fluorescence under constant illumination, is another factor that impairs the SNR, especially during long recording sessions. Photostable probes exhibit low to moderate bleaching during an experiment and do not require extensive bleach-correction to isolate the signals. GFP-based indicators tend to photobleach faster than opsins, a limitation attributable to the properties of currently available fluorophores.

The spectral properties of an FP also have an impact on the SNR for *in vivo* imaging. Optical signals, especially from blue-shifted probes (λ < 650 nm) are prone to attenuation from absorption and scattering in the brain and exhibit low contrast amid green tissue autofluorescence. Red-shifted probes are less subject to loss from absorption or scattering and the attenuation coefficient at 600–700 nm is about a half of that at 500 nm. As a result, their optical transmittance (tissue penetration) is almost twice as high (Flock et al., 1992; Al-Juboori et al., 2013).

One should bear in mind, however, that the optical and spectral properties of an FP may not apply to an engineered GEVI because fusing an FP with VSD and mutating its primary sequence can influence protein folding and chromophore environment to substantially modify the native fluorophore.

#### Probe Expression

Exclusive expression of GEVIs in the plasma membrane is important to minimize contamination of useful signals with intracellular fluorescence refractory to voltage changes. To some extent, this can be achieved by inserting membrane-trafficking signals into the coding sequence or coopting pH-sensitive fluorophores, such as ecliptic pHluorin and SEP, whose fluorescence is “eclipsed” in acidic intracellular compartments. In addition, adequate transgene expression requires the use of appropriate vectors for gene delivery such as recombinant adeno-associated viruses (rAAVs) or plasmid DNA for *in utero* electroporation.

It is also important to achieve sparse expression of indicators in select neurons to reduce background fluorescence from dampening the SNR. For some types of neurons such as cortical pyramidal cells and interneurons, layer-specific expression can be achieved by delivery of plasmid DNA to embryos carried by timed-pregnant mice via *in utero* electroporation (Wang and Mei, 2013; De Marco Garcia and Fishell, 2014). Specific neuronal subtypes can also be targeted by exploiting differences in their gene expression profiles. Using cell-type specific promoters together with combinatorial gene targeting strategies such as Cre recombinase/loxP or tetracycline transactivation (tTA) systems, it is possible to both spatially and temporally regulate indicator expression.

#### Optical Instrumentation

As with any fluorescence microscopy, the SNR in functional imaging also depends on the optical setup.

High numerical aperture (NA) (∼1.2) objectives can deliver more excitation photons and collect more fluorescent photons, thereby enhancing signal brightness and resolution [resolvable distance *R* = λ/ (2 × *NA*)] (Piston, 1998). When coupled with high-resolution detectors, they enable better identification of ROIs. Illumination intensities should be adequate to overcome shot-noise limitations from stochastic fluctuations in photon counts. Yet, the light intensity should not be overpowering as it can accelerate photobleaching, cause sample heating and phototoxicity. Likewise, emission filters should be carefully chosen to minimize fluorescence contamination with excitation light, which can drastically obscure the signals. FPs with large Stokes shift – the difference between the absorption and emission maxima – are useful for avoiding spectral overlap (Piatkevich et al., 2010, 2013; Chu et al., 2016).

The choice of detector is another factor that can influence the SNR. CCD cameras such as those offered by RedShirt Imaging, LLC are particularly suited for high speed imaging at up to 2 kHz, or 5 kHz with 3 pixel × 3 pixel binning, and enable detection of optical responses to APs. Given their high quantum efficiency (>80%) – a measure of the percentage of photons detected – and low read noise, these cameras provide SNRs close to the theoretical maximum.

Although conventional one-photon microscopy provides fast acquisition rates because photons are sampled in parallel, two-photon laser scanning is a better option for *in vivo* imaging as it provides superior SNR due to three-dimensional optical sectioning and enhanced spatial resolution. Type 1 VSD-based indicators such as ArcLight and ASAP2f have been successfully used for imaging slow, subcellular voltage dynamics *in vivo* (Cao et al., 2013; Storace et al., 2015; Yang et al., 2016). For instance, ArcLight reports odorant responses in populations of olfactory glomeruli with a sensitivity of about -1 to -2% Δ*F*/*F*_0_ in single trial recordings under two-photon microscopy as with widefield imaging (Storace et al., 2015). However, the large pixel dwell times of contemporary lasers and low frame rates (10–100 Hz) preclude their use in imaging responses to rapid voltage fluctuations viz. APs (<1–2 ms duration). To overcome this issue, random access scanning, which permits fast acquisition rates of up to 1 kHz, has recently been demonstrated to work well with a novel ASAP variant ASAP2s. Nevertheless, while the large sensitivity of ASAP2s (-15% Δ*F*/*F* per AP under widefield imaging conditions) was recapitulated under two-photon microscopy, the probe suffered more profoundly from photobleaching during laser scanning compared to one-photon imaging (Chamberland et al., 2017).

It is unclear whether opsin-based GEVIs are optimized for two-photon imaging. Recent studies have demonstrated that opsin GEVIs such as Arch-based indicators, Ace-mNeon and MacQ-mCitrine perform poorly under laser scanning microscopy. A prevailing hypothesis is that the femtosecond pulsed laser likely interferes with rhodopsin photocycle and opsin GEVIs may not be held in their voltage-sensitive state during the “dark” phase (Brinks et al., 2015; Chamberland et al., 2017).

As an alternative imaging modality, a powerful fiber-optic technique has recently been shown to enable cell-type specific recordings of transmembrane voltage dynamics using opsin-FRET indicators. The 0-D recording technique, dubbed Transmembrane electrical measurements performed optically or TEMPO, allows for fluorescence activation by laser beams delivered via optic fibers and signal detection by photoreceivers coupled to lock-in amplifiers. The technique offers a sensitivity approaching theoretical limits imposed by photon-shot noise (Marshall et al., 2016).

One could, in principle, use high-speed epifluorescence imaging to record spikes *in vivo* but the data will require heavy post-processing to distinguish voltage responses from background fluorescence. Besides, the images will still provide little spatial information, which can only be inferred from the processed signals. In the study describing Ace2N-mNeon, optical responses to spontaneous firing *in vivo* were captured using wide-field imaging at up to 2 kHz acquisition rate. The neurons were located by the electrical activity from a juxtacellular electrode. The images were subjected to background subtraction and voltage responses were selectively extracted from pixels that exhibited the top 20% SNR values, which mostly corresponded to the somata of interest (Gong et al., 2015).

### Response Kinetics

Improving the speed of voltage indicators to follow individual APs and resolve fast spike trains can maximize their usefulness in neuroscience research. However, as mentioned above, advances in imaging modalities must complement GEVI engineering directed at improving response kinetics.

**FIGURE 5:**
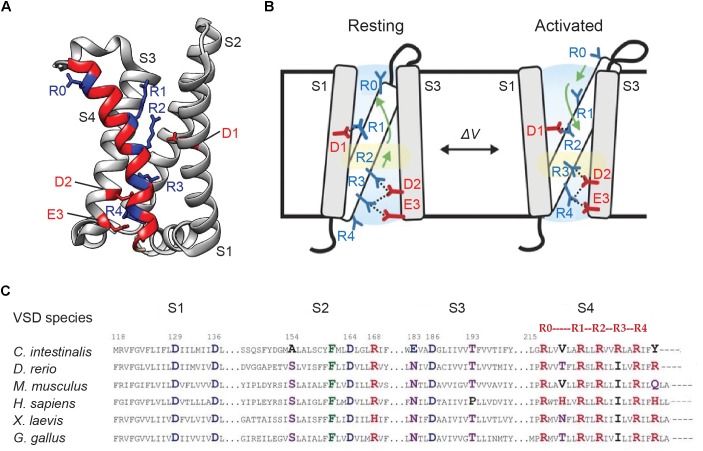
Genetically encoded voltage indicators kinetics and voltage dependence of optical responses largely depend on voltage-sensitive conformational changes in its VSD. **(A)** Crystal structure of the VSD of *Ciona intestinalis* voltage sensitive phosphatase (PDB ID 4G7V). The four transmembrane helices S1–S4 are shown. S4 is highlighted in red. The position of the gating arginines R1–R4 (every third amino acid starting at 223) as well as the outermost S4 arginine R0 (position 217 in *Ci*VSD) are shown in blue. The countercharges D1 (on S2 helix), D2 and E3 (on S3 helix) are indicated in red. **(B)** A mechanistic model of voltage sensing in *Ci*VSD. The activated UP state is accompanied by a 5 Å S4 down movement and a ∼60° counterclockwise rotation of the entire helix. The arginines are then stabilized by successive negative countercharges, from R1–D1, R2–D2, and R3–D3 pairings in the resting or DOWN state to R2–D1, R3–D2, and R4–E3 in the UP configuration. **(C)** Sequence alignment of the VSD transmembrane segments of voltage sensitive phosphatase orthologs. Positive, negative and polar residues are indicated in red, blue, and purple, respectively. The numbering follows that of the *Ciona* homolog (Li et al., 2014; Piao et al., 2015).

In ArcLight and other VSD-based GEVIs, the kinetics is largely controlled by voltage-sensing movements of the VSD. In response to voltage fluctuations, VSDs exhibit “gating” charge movements, which as part of native ion channels and voltage-sensitive phosphatases, facilitate channel gating or enzyme activation. Structural studies show that voltage-activation of *Ci*VSD causes the S4 helix to slide upward by ∼5 Å along its main axis, and rotate by ∼60°, to mobilize the putative gating charges – a series of evenly-spaced arginines (Mannuzzu et al., 1996; Yang et al., 1996; Li et al., 2014) (Figure 5A,B). This conformational change is hypothesized to pervade the FP in voltage indicators, thereby modulating their fluorescence output.

In contrast, the voltage sensitivity of opsin-based indicators does not depend on movements of the sensor but instead on changes in the protonated state of the chromophore retinal. These changes are thought occur near instantaneously during voltage transients, and as result, most standalone opsins and opsin-FRET GEVIs exhibit accelerated kinetics in the sub-millisecond timescales.

#### Source of VSD

Fast kinetics was first observed in the ion channel-based voltage indicator SPARC. SPARC consists of rat skeletal sodium channel rNav1.4 fused to eGFP, which is inserted in the intracellular loop between its second and third domains. The fast kinetics of the fluorescence response (exponential rise time τ-on <1 ms; <0.5% Δ*F*/*F*_0_ per 100 mV) reflects the fast voltage-dependent gating charge movements, likely in transmembrane domains I and II (Ataka and Pieribone, 2002).

Most *Ci*VSD-based probes have slow rise times lasting tens of milliseconds and slower decays of about 80–100 ms (Dimitrov et al., 2007; Tsutsui et al., 2008; Perron et al., 2009; Jin et al., 2012). ArcLight-Q239, the variant with the shortest linker and maximum sensitivity, exhibits double exponential kinetics with on-times of 9 (τ1) and 48 (τ2) ms, and off-times of 17 (τ1) and 60 (τ2) ms (per 100 mV). The fast components (τ1s) during rise and decay make up 50 and 80% of the total amplitude, respectively.

Several approaches have been shown to alter the kinetics of VSD-based probes. One of them has been to simply replace *Ci*VSD with orthologs. Substituting *Dr*VSD or *Gg*VSD, for instance, generates ArcLight variants with faster and simpler kinetics (single exponential) with τ-on and τ-off time constants of ∼5 and ∼9 ms, respectively. These variants, however, exhibit reduced sensitivities, compared to the *Ci*VSD variant, presumably due to altered interactions between the VSD orthologs and SEP 227D. Nevertheless, the chicken ArcLight can detect APs in cultured neurons in single trials with a sensitivity of ∼-3% Δ*F*/*F*_0_ and resolve simulated APs at 100 Hz in HEK cells (Han et al., 2013).

The zebrafish and chicken VSDs similarly boost response kinetics as part of other GEVI scaffolds. For instance, Zahra, a fusion between *Dr*VSD and CFP/YFP FRET pair, exhibits time constants of ∼3.5–5 ms per 100 mV depolarization, a significant improvement over its predecessor *Ci*VSD-based VSFP2.1 (Dimitrov et al., 2007; Baker et al., 2012). Similarly, another FRET-based sensor Mermaid, displays improved kinetics that compares to *Gg*VSD-ArcLight when *Ci*VSD is replaced with the chicken ortholog (Tsutsui et al., 2008; Han et al., 2013). *Gg*VSD also imparts superior kinetics to ASAP1/2, in which a cp-superfolder GFP is inserted in the extracellular S3–S4 loop. Although ASAP1/2 display double exponential kinetics, the responses are dominated by fast components (τ1 = 2 ms) (St-Pierre et al., 2014).

**FIGURE 6:**
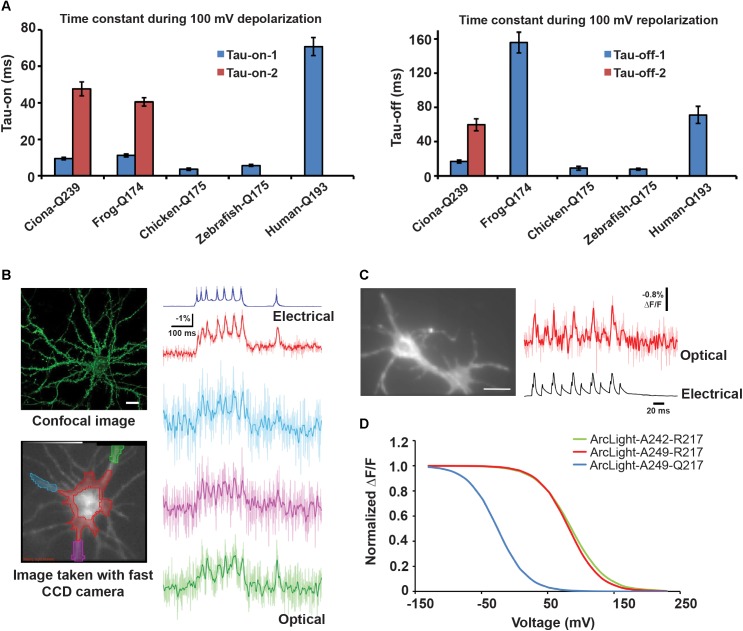
Changing the VSD species or circular permutation of FP enhances GEVI kinetics **(A)** τ-on and τ-off of optical responses to 100 mV depolarization in HEK cells transfected with ArcLight derivatives based on frog, chicken, zebrafish or human voltage sensitive phosphatase ortholog. **(B)** Top left: confocal image of cultured hippocampal neuron expressing chicken ArcLight. Scale bar = 20 μm. Bottom left: An 80 × 80 fast CCD camera image of a neuron from which electrical and optical recordings in the right panel were obtained. The ROIs are color coded to match those of the respective optical traces. Right: Single trial recordings of evoked APs using chicken ArcLight. The somatic and neurite recordings were low-pass filtered with a 95 and 50 Hz Gaussian filter, respectively. The unfiltered traces are shown as light-colored lines. **(C)** Left: Wide-field image of a hippocampal neuron expressing ElectricPk. Scale bar = 15 μm. Right: Single (light red trace) and averaged (red trace) optical and electrical responses to evoked APs in the neuron on the left. The optical responses were captured using a NeuroCCD camera (RedShirt Imaging, LLC) at 2 kHz. **(D)** Normalized Δ*V*/Δ*F* curves for ArcLight derivatives A242–R217, S249–R217, and S249–Q217. 242 and 249 represent sites of FP fusion on the *Ci*VSD linker. 217 refers to the outermost arginine on the S4 helix (Barnett et al., 2012; Han et al., 2013, 2014).

In contrast, frog and human VSDs impair ArcLight’s kinetics by generating responses slower than the *Ciona* version (τ-on = 11 and 70 ms, τ-off = 155 and 70 ms, respectively) (Figure 6A,B). It is intriguing why different VSD orthologs exhibit differential kinetics. In part, the discrepancies appear to stem from variations in their primary sequence, which may affect the speed of gating charge movements.

Sequence alignment of VSD orthologs from 29 species reveals that while several residues across the helices are highly conserved, with strikingly similar arginine repeats in S4, conspicuous differences exist at some of these sites. Combinatorial mutations of the variable residues in *Ci*VSD (Figure 5C) led to the development of the fast SEP/227D-containing sensor Bongwoori. The *Ci*VSD in Bongwoori contains mutations in S2 and S4 helices (A154D/R229I), which enhance its kinetics, as well as the S4 mutation R217Q, which augments sensitivity to negative potentials. The probe exhibits a similar τ-on (∼8 ms) as ArcLight, but the fast component constitutes a larger fraction of the total exponential decay (91% versus 50%). Its τ-off is 7 ms (Piao et al., 2015).

#### Circular Permutation of FP

Voltage indicator kinetics are not only limited by movements of the voltage sensor but also the efficiency and speed of coupling between the sensor and the fluorophore during these movements.

Circular permutation of the FP, regardless of VSD species, enhances response kinetics. ElectricPK, a fast voltage sensor, was discovered during a screen for voltage sensitivity among a series of *Ci*VSD-cp-eGFP scaffolds. This probe, and in fact many other candidates from the screen, exhibited fast on and off rates of ∼2 ms. ElectricPK can reliably resolve short, high frequency depolarization pulses in HEK cells, and APs in hippocampal neurons, with discrete optical responses, unobscured by accumulating baseline drift (Barnett et al., 2012) (Figure 6C).

Similarly, the *Ci*VSD-cp-mApple fusion protein FlicR1 exhibits fast kinetics (τ1 = ∼3 ms), with responses dominated by fast components. FlicR1 was developed by random mutagenesis and contains eight mutations in its VSD. It is hence unclear whether the increase in speed is an outcome of circular permutation alone or additional changes to the kinetics of the VSD (Abdelfattah et al., 2016). This is also the case for ASAP1/2, where both VSD species and FP assembly are propitious to accelerated voltage-sensing.

Upon voltage activation, *Ci*VSD appears to undergo multiple structural rearrangements. While the initial response is fast and involves the gating charge movement, it is followed by other slower and plausibly voltage-independent transitions. These two components of VSD dynamics are mirrored in the FP and hence the GEVI’s fluorescence output (Kohout et al., 2008; Villalba-Galea et al., 2009). The consensus is that FPs of slower probes reminisce both types of VSD movements whereas those of faster probes capture only the former. Circular-permuted FPs, in particular, effectively couple with the fast movements as they are more vulnerable to barrel distortions and rapidly destabilize given their sub-optimal configuration.

#### Kinetics of the Ideal Voltage Sensor

Given that APs are rapid events, one may predict that a GEVI with sub-millisecond kinetics can adeptly resolve a quick succession of APs, providing precise information on both spike incidence and timing. Indeed, opsin-based GEVIs with sub-millisecond kinetics, arising from retinal state transitions of microsecond time-scales, provide superior temporal resolution for spike detection (Maclaurin et al., 2013; Gong et al., 2014, 2015; Zou et al., 2014). However, spike detection fidelity for a probe *d′* has been shown to depend not on the temporal accuracy of overlap between the optical signal and AP waveform, but rather on the duration of the optical signal, even if it exceeds that of the underlying spike (Wilt et al., 2013).

Under photon shot-noise limited conditions, contemporary detectors offer *d′* that is dependent on signal size Δ*F*/*F*, brightness of the sensor *F*_0_ and signal decay time τ such that $d^{\prime }\approx (\Delta F/F)•\sqrt{(F_{0}\text{τ})/2}$ (Wilt et al., 2013). In addition to improving the sensitivity and brightness of the probes, prolonging the decay time of the optical signal improves *d′*, as it allows for additional signal photons to be collected per event, above background. Thus, an optical indicator with a swift rise capable of precisely sensing spike onset, but protracted decay, can effectively improve spike detection. Nevertheless, such indicators may fail to provide accurate spike timing information, when spikes arrive at frequencies greater than the resolvable limit of the indicator.

In sum, when choosing an indicator for its response kinetics, one may want to consider experimental priorities such as whether a given biological question warrants precise spike detection, spike timing estimation or assessment of subthreshold voltage changes. While fast GEVIs may be well-suited for the former type of questions, slower GEVIs may be valuable tools for studying input patterns in local circuits, such as voltage changes from synaptic transmission, and slow population-wide voltage oscillations.

### Voltage Dependence of Optical Responses

The overarching goal of GEVI engineering is to develop optical indicators that are sensitive to both depolarization and hyperpolarization, provide absolute measures of voltage change and operate in the physiological voltage range for neurons (voltage of half-maximal fluorescence response *V*_1/2_ ≈ -70 mV). All of these can be achieved by optimizing a probe’s Δ*V*/Δ*F* curve.

The properties of the Δ*V*/Δ*F* curve are also determined by a GEVI’s voltage sensing motif. Since native VSDs are activated by membrane depolarization, the voltage dependence of gating charge movements is shifted toward positive potentials. This means that GEVIs incorporating wild type VSDs would operate only at highly depolarizing voltages. However, it is possible to expand a VSD’s sensitivity range to include less depolarizing and even hyperpolarizing voltages. For example, in the *Shaker* potassium channel, neutralization of the outermost positive charge in S4 (R362Q) produces gating charge movements even at voltages below -70 mV (hyperpolarization) (Seoh et al., 1996). Analogous mutations in phosphatase-derived VSDs similarly broaden their sensitivity enhancing the usefulness of GEVIs over a wide range of voltages.

The *Ci*VSD mutation R217Q, and the corresponding *Gg*VSD and *Dr*VSD mutations (R153Q), increase GEVI sensitivity to hyperpolarizing potentials and result in leftward shifts of the Δ*V*/Δ*F* curve (Figure 6D). Accordingly, these mutations have been widely adopted in a number of probes (Dimitrov et al., 2007; Tsutsui et al., 2008; Baker et al., 2012; Han et al., 2013, 2014; Piao et al., 2015). Neutralization of a countercharge (D164) in the S2 helix also enhances responsiveness to negative voltages (Piao et al., 2015). By increasing sensitivity to hyperpolarization, these mutations serve to place the *V*_1/2_ closer to the resting membrane potential. In practice, however, *V*_1/2_ is highly variable across GEVIs and in many probes, still leans toward depolarization (∼-35 to -3 mV) (Tsutsui et al., 2008; Jin et al., 2012; Piao et al., 2015). The discrepancy suggests that additional factors such as the coupling between the VSD and FP may play a role in determining the voltage dependence of optical responses.

Aside from negative shifts, the slope of the Δ*V*/Δ*F* curve is another indicator of a probe’s sensitivity across different voltages. For instance, a probe with a large slope that exhibits a large Δ*F* for small voltage fluctuations, may be useful for monitoring subthreshold voltage changes, whereas, a GEVI with a smaller slope, showing the same absolute Δ*F* but upon larger voltage changes may be appropriate for detecting spikes. At least one factor that seems to affect the slope of the Δ*V*/Δ*F* curve is the presence of charged residues in S2 (Piao et al., 2015). However, further studies are required to better understand the voltage dependence of optical responses to be able to better tailor GEVIs to meet specific experimental needs.

## Conclusion

In the two decades since their discovery, we have begun to understand the challenges associated with GEVI engineering and are striving toward making better probes with enhanced membrane expression, voltage sensitivities and SNR, while also tailoring their speed and dynamic voltage range to fulfill experimental needs. Because each GEVI comes with its own unique advantages and serves distinct purposes in the laboratory, it may be hard to envision an ideal probe that combines the best qualities of all existing probes and provides a unanimous solution for all experimental demands. The goal of GEVI development instead should be to capitalize on a given probe’s potential and maximize its utility by strategic and rigorous engineering.

Genetically encoded voltage indicators engineering involves at least three steps – development, delivery and diagnosis. The development starts with analyzing protein crystal structures, where available, and sifting through large volumes of literature to design a preliminary scaffold. The genetic sequence of the putative voltage indicator then needs to be engineered by directed evolution, and the mutants tested iteratively for their performance. While in the past, low to moderate-throughput efforts have focused on manual hierarchical screening for brightness and voltage responsiveness, significant strides have been made to automate these processes and improve throughput. It is now possible to democratize voltage engineering using robotic systems and serially optimize millions of variants for multiple parameters such as brightness, membrane localization and voltage sensitivity (Piatkevich et al., 2018). Likewise, in our laboratory, we have developed semi-automated microscopic systems that are equipped with field electrodes and sensitive detectors to simultaneously optimize indicator constructs in a 96-well plate format for voltage responses, brightness and localization in high throughput and high content (Kannan et al., 2018).

Once a probe is adequately optimized for reporting neuronal activity and characterized in cell culture, the next step is to deliver it in animals to monitor its usefulness in intact circuits *in vivo.* This can be accomplished by injecting recombinant viruses or electroporating plasmid DNA, encoding the engineered GEVI. The choice of promoter, use of inducible regulators of gene expression, and timing of gene delivery can target indicator expression to specific cell types at defined stages of development.

The last step is diagnosis. Although much of the innovation happens early on during discovery, diagnosis is perhaps the most creative phase of GEVI development. It involves designing an experimental paradigm which allows one to explore the entire gamut of applications where an indicator may be of use. A probe may be used as a reporter of spontaneous or stimulus-evoked activity, developmental or experience-dependent plasticity, learning and memory or other forms of behavior. The surgical and optical setup may also need to be customized for the probe. Novel red and far-red indicators may need to be adequately demonstrated for their ability to be multiplexed with blue-shifted optogenetic tools and other types of indicators with minimal optical cross-talk.

Genetically encoded voltage indicators engineering requires a revisit to the fundamentals of voltage-dependent mechanisms, distinct from cellular calcium signaling, and an understanding and appreciation of the advantages of voltage probes to inspire us during the arduous process of innovation. Considering the steady advances in probe engineering and anticipated developments in imaging technologies, we are probably closer to our destination now than ever before.

## Author Contributions

MK, GV, and VP wrote the manuscript. MK and GV prepared the figures.

## Conflict of Interest Statement

The authors declare that the research was conducted in the absence of any commercial or financial relationships that could be construed as a potential conflict of interest.
